# Filgotinib Improved Health-Related Quality of Life and Led to Comprehensive Disease Control in Individuals with Ulcerative Colitis: Data from the SELECTION Trial

**DOI:** 10.1093/ecco-jcc/jjad018

**Published:** 2023-06-16

**Authors:** Stefan Schreiber, Brian G Feagan, Laurent Peyrin-Biroulet, Séverine Vermeire, Margaux Faes, Kristina Harris, Alessandra Oortwijn, Patrick Daniele, Haridarshan Patel, Silvio Danese

**Affiliations:** University Hospital Schleswig-Holstein, Kiel, Germany; Alimentiv Inc., London, Ontario, Canada; Western University, London, Ontario, Canada; Department of Gastroenterology, University of Lorraine, CHRU-Nancy, Nancy, France; University of Lorraine, Inserm, NGERE, Nancy, France; Department of Gastroenterology and Hepatology, University Hospitals Leuven, KU Leuven, Leuven, Belgium; Galapagos NV, Mechelen, Belgium; Galapagos NV, Milan, Italy; Galapagos NV, Leiden, Netherlands; Cytel Health Canada, Toronto, Canada; Galapagos NV, Mechelen, Belgium; Gastroenterology and Endoscopy, IRCCS Ospedale San Raffaele, Milan, Italy; Vita-Salute San Raffaele University, Milan, Italy

**Keywords:** Clinical trials, quality of life, socio-economical and psychological endpoints

## Abstract

**Background and Aims:**

Ulcerative colitis [UC] impacts patients’ health-related quality of life [HRQoL]. We assessed HRQoL and an exploratory patient-level composite endpoint (‘Comprehensive Disease Control’ [CDC]) in individuals receiving filgotinib [an oral JAK1 preferential inhibitor] in the SELECTION trial.

**Methods:**

In SELECTION [NCT02914522], a double-blind, randomized, placebo-controlled, phase 2b/3 trial, adults with moderately to severely active UC received once-daily filgotinib 200 mg, filgotinib 100 mg or placebo for 11 weeks in Induction Study A [biologic-naïve] or B [biologic-experienced]. Filgotinib responders [week 10 clinical remission/response] were re-randomized to their filgotinib regimen or placebo for the 48-week Maintenance Study. We assessed week 10 and week 58 SF-36, EQ-5D, WPAI and IBDQ scores. Achievement of CDC (patient-level partial Mayo Clinic Score [pMCS] remission [pMCS ≤2, no individual rectal bleeding, stool frequency or physician’s global assessment subscore >1], endoscopic improvement [endoscopic subscore ≤1], faecal calprotectin <150 µg/g and IBDQ score ≥170) and its association with HRQoL and histological outcomes were also explored.

**Results:**

Analyses included 382 biologic-naïve and 404 biologic-experienced patients. Filgotinib 200 mg induced and maintained improvements vs placebo in SF-36, EQ-5D, WPAI and IBDQ scores, and restored HRQoL by week 10. Proportionally more filgotinib 200 mg- than placebo-treated patients achieved CDC at weeks 10 and 58 [*p* < 0.01]. CDC was associated with clinically important improvements in HRQoL and histological remission over both periods.

**Conclusions:**

Filgotinib 200 mg results in short- and long-term improvements in HRQoL. High-level improvement of HRQoL relates to a stringent composite endpoint suggesting meaningful disease control in a subset of filgotinib-treated individuals.

ClinicalTrials.gov identifier: NCT02914522

## 1. Introduction

Ulcerative colitis [UC] is an inflammatory bowel disease of the colonic mucosa that negatively affects patients’ health-related quality of life [HRQoL].^1,2^ Approximately half of patients develop a complex course of disease, with chronic activity or frequent relapses, despite advanced therapy.^3^ Common UC symptoms, including diarrhoea, haematochezia, abdominal pain, urgency, tenesmus, anaemia and fatigue, are important determinants of HRQoL that negatively affect psychological, physical, sexual and social functioning.^1,4–8^ Reduced HRQoL is in turn one of the strongest factors affecting work productivity loss among employed patients with UC.^9^

Patients consider the ability to improve HRQoL to be among the most important attributes of a UC treatment.^10^ Accordingly, long-term treatment goals now include improvement/normalization of HRQoL, in addition to conventional targets such as the induction and maintenance of clinical remission, endoscopic mucosal healing and symptom relief.^11^ Although most clinical trials conducted in patients with UC evaluate each of these outcomes, they are often assessed independently of one another, and at the level of the overall patient population, rather than as composite measures at the patient level. While these global assessments are useful for assessing the overall efficacy of a therapy, a composite, patient-level endpoint would be a valuable tool for clinicians in assessing the diverse targets specified by treatment guidelines [including HRQoL measures related to the patient’s function and well-being,^12^ as well as symptoms and objective disease activity].

Filgotinib is an oral Janus kinase [JAK] 1 preferential inhibitor that has been approved in the European Union and the UK for the treatment of adults with moderately to severely active UC.^13,14^ In the phase 2b/3 SELECTION trial, filgotinib 200 mg was efficacious in inducing and maintaining remission in biologic-naïve and biologic-experienced patients with UC.^15^

In these exploratory and *post hoc* analyses of SELECTION data, we examined the effect of filgotinib on generic HRQoL (measured using 36-Item Short-Form Survey [SF-36] and EuroQol 5-dimension [EQ-5D] scores), disease-specific HRQoL (measured using the Inflammatory Bowel Disease Questionnaire [IBDQ] score) and work productivity (measured using Work Productivity and Activity Impairment [WPAI] questionnaire scores), and examined the effect of filgotinib on restoration of generic HRQoL, as assessed using SF-36 scores. In addition, we explored disease control at the patient level by evaluating achievement of an exploratory composite endpoint, herein referred to as ‘Comprehensive Disease Control’ [CDC], comprising objective measures of disease activity and patient-reported outcomes. Finally, we assessed the correlation of this endpoint with generic HRQoL, work productivity scores and histological remission.

## 2. Materials and Methods

### 2.1. Overall study design

SELECTION [NCT02914522] was a combined phase 2b/3 double-blind, randomized, placebo-controlled trial comprising two induction studies and a Maintenance Study. Details of the study design have been previously described by Feagan *et al*.^15^ Eligible patients with moderately to severely active UC (Mayo endoscopic subscore ≥2, rectal bleeding subscore ≥1, stool frequency subscore ≥1, physician’s global assessment subscore ≥2; total Mayo Clinic Score [tMCS] of 6–12) were enrolled into one of two induction studies: biologic-naïve patients entered Induction Study A and biologic-experienced patients entered Induction Study B. Patients were randomized 2:2:1 to receive filgotinib 200 mg, filgotinib 100 mg or placebo orally once daily for 11 weeks. Those who achieved either clinical remission or a tMCS response at week 10 [responders] were re-randomized 2:1 at week 11 to continue their induction filgotinib regimen or receive placebo in the Maintenance Study through week 58. Placebo responders continued to receive placebo. Clinical remission was defined as a Mayo endoscopic subscore of 0 or 1, a rectal bleeding subscore of 0 and at least a 1-point decrease in stool frequency subscore from induction baseline to achieve a subscore of 0 or 1. tMCS response was defined as a reduction of at least 3 points in tMCS and at least 30% from induction baseline, with an accompanying decrease in rectal bleeding subscore of at least 1 point, or an absolute rectal bleeding subscore of 0 or 1. Full details of the inclusion and exclusion criteria for enrolment into the induction and maintenance studies have been given by Feagan *et al*.^15^ Week 10 non-responders were excluded from the Maintenance Study but could enter the ongoing long-term extension study, SELECTIONLTE [NCT02914535]. Each study was carried out in accordance with the International Conference on Harmonisation Good Clinical Practice Guidelines and the Declaration of Helsinki. All patients provided written informed consent before any study procedures were carried out.

### 2.2. Pre-specified exploratory analyses

As part of pre-specified exploratory analyses of SELECTION, changes from induction/maintenance baseline in the following outcomes were assessed at weeks 10 and 58: generic HRQoL, as measured by SF-36 physical component summary [PCS], mental component summary [MCS] and subscale scores, as well as EQ-5D visual analogue scale [VAS] and EQ-5D 5-level [-5L] UK utility scores; and disease-specific HRQoL, as measured by IBDQ total score and subscores.

### 2.3. *Post hoc* analyses

#### 2.3.1. Achievement of MCIDs in HRQoL outcomes

The proportions of patients achieving the minimal clinically important difference [MCID] in the following measures at weeks 10 and 58 were assessed: SF-36 PCS score, SF-36 MCS score, EQ-5D VAS score, EQ-5D UK utility score, IBDQ total score, combined IBDQ and SF-36 scores [achievement of the MCID in each of IBDQ total score, SF-36 PCS score, and SF-36 MCS score], and WPAI activity impairment score. In addition, the proportions of patients who were in employment at induction baseline and who achieved the MCID in WPAI absenteeism, presenteeism and work productivity loss scores were assessed at weeks 10 and 58. EQ-5D-5L values were standardized to the EQ-5D 3-level [-3L] according to the algorithm proposed by van Hout *et al*.,^16^ and scored using the corresponding EQ-5D-3L UK value set to derive health utilities based on the UK population.^17^ Full details of each of the HRQoL outcome measures, including MCID thresholds, are given in Supplementary Table 1.

#### 2.3.2. Restoration of SF-36-defined HRQoL

SF-36 scores relative to age–sex norms were examined. The proportions of patients with an SF-36 PCS or MCS score <40 at induction baseline who achieved restored SF-36-defined HRQoL [SF-36 PCS or MCS score ≥40]^18^ at week 10, and the proportion of patients who maintained normal HRQoL at week 58, were evaluated.

#### 2.3.3. Assessment of individual patient treatment benefits: achievement of an exploratory composite endpoint, CDC

The proportion of patients who achieved CDC, comprising four established treatment outcomes, was assessed at weeks 10 and 58. Patients were considered to have achieved CDC if they achieved: (1) partial Mayo Clinic Score [pMCS] remission [pMCS ≤2 and no individual rectal bleeding, stool frequency or physician’s global assessment subscore >1]; (2) endoscopic improvement [Mayo endoscopic subscore of 0 or 1]; (3) inflammatory biomarker remission [faecal calprotectin <150 µg/g]^19^; and (4) IBDQ remission [IBDQ total score ≥170]. The proportions of patients who achieved minimal clinically important improvements from induction baseline to week 10 and minimal clinically important declines from maintenance pre-baseline [week 11] to week 58 in SF-36, EQ-5D and WPAI scores were assessed among those who achieved CDC and those who did not. In addition, the proportions of patients in histological remission [Geboes score ≤2B.0] within each group were assessed at weeks 10 and 58.

### 2.4. Statistical analyses

These analyses were conducted using the SELECTION induction and maintenance full analysis sets.^15^ For the induction studies, the full analysis sets included all randomized patients who received at least one dose of the study drug within that study. For the Maintenance Study, the full analysis set included all patients randomized to either filgotinib 200 mg or filgotinib 100 mg in the induction studies who achieved clinical remission or a tMCS response at week 10, and who were re-randomized in the Maintenance Study [and received at least one dose of the study drug in the Maintenance Study].

Patient demographics and baseline characteristics were analysed using descriptive statistics. Changes from induction baseline to week 10 and from maintenance baseline to week 58 were calculated for each HRQoL measure using least-squares [LS] mean changes with 95% confidence intervals [CIs]. Differences in LS mean changes were estimated using analysis of covariance adjusted for baseline HRQoL measures and stratification factors. Treatment assignments and analyses were stratified in Induction Study A by day 1 use of oral systemic corticosteroids and use of immunosuppressants [6-mercaptopurine, azathioprine and methotrexate]; in Induction Study B, by the same factors as in Induction Study A, and by previous exposure to one vs more than one biologic; and in the Maintenance Study, by the same factors as in Induction Study A, and by participation in Induction Study A or B. Binary assessment of the proportion of patients achieving the MCID in each of the HRQoL measures at weeks 10 and 58 was performed using Cochran–Mantel–Haenszel tests. No adjustment for multiple comparisons was made. A logistic regression model was used to calculate odds ratios with 95% CIs for restoration of SF-36-defined HRQoL at week 10 and maintenance of normal SF-36-defined HRQoL at week 58 for filgotinib 200 mg compared with placebo. The proportions of patients who achieved CDC at weeks 10 and 58, and the proportions of patients who had minimal clinically important improvements and declines in HRQoL measures from induction baseline to week 10 and from maintenance baseline to week 58, respectively, among CDC achievers and non-achievers, were compared using Pearson’s chi-squared test.

All missing data were imputed using a last observation carried forward approach, except for the proportions of patients in IBDQ remission, for which non-responder imputation was used. Because these were exploratory and *post hoc* analyses, all *p* values are considered nominal.

## 3. Results

### 3.1. Patient disposition and baseline characteristics

These analyses included 382 and 404 patients from Induction Studies A and B, respectively, and 297 patients from the Maintenance Study. Patient baseline demographic and disease characteristics were similar between treatment groups within each induction study [Table 1]. In addition, mean SF-36, EQ-5D, IBDQ and WPAI scores were similar across treatment groups and studies at induction baseline, with mean IBDQ total scores of <170.

**Table 1.** Baseline demographics and characteristics of patients in Induction Studies A and B

	Induction Study A: biologic-naïve patients		Induction Study B: biologic-experienced patients	
	Placebo	Filgotinib 200 mg	Placebo	Filgotinib 200 mg
	[*n* = 137]	[*n* = 245]	[*n* = 142]	[*n* = 262]
Age, years, mean ± SD	41 ± 12.9	42 ± 13.1	44 ± 14.9	43 ± 14.2
Sex, female at birth, *n* [%]	50 [36.5]	122 [49.8]	56 [39.4]	114 [43.5]
Race,^a^*n* [%]				
Asian	38 [27.7]	77 [31.4]	27 [19.0]	50 [19.1]
Black or African-American	1 [0.7]	2 [0.8]	3 [2.1]	4 [1.5]
White	95 [69.3]	165 [67.3]	98 [69.0]	190 [72.5]
Duration of UC from diagnosis, years, mean ± SD	6.4 ± 7.4	7.2 ± 6.9	10.2 ± 8.2	9.8 ± 7.6
Mayo Clinic Score, mean ± SD	8.7 ± 1.3	8.6 ± 1.3	9.3 ± 1.4	9.2 ± 1.4
Endoscopy subscore of 3, *n* [%]	76 [55.5]	133 [54.3]	111 [78.2]	203 [77.5]
HsCRP, mg/L, mean ± SD	5.8 ± 7.6	8.6 ± 16.3	14.0 ± 24.3	12.2 ± 14.9
Faecal calprotectin, μg/g, mean ± SD	2231 ± 2916.9	2059 ± 2639.1	2479 ± 3571.4	2845 ± 4076.5
Concomitant use of corticosteroids on day 1,^b^* n* [%]	34 [24.8]	54 [22.0]	51 [35.9]	94 [35.9]
Concomitant use of immunomodulators on day 1,^b^* n* [%]	33 [24.1]	53 [21.6]	21 [14.8]	34 [13.0]
SF-36 score, mean ± SD				
PCS	42.5 ± 6.9	42.2 ± 6.8	40.1 ± 8.1	40.6 ± 7.8
MCS	37.7 ± 9.5	39.5 ± 9.5	39.9 ± 10.3	37.9 ± 10.9
EQ-5D score, mean ± SD				
VAS	51.8 ± 19.1	53.7 ± 18.9	49.0 ± 18.9	48.1 ± 20.5
5L UK utility	0.680 ± 0.190	0.697 ± 0.165	0.636 ± 0.216	0.615 ± 0.252
WPAI score, mean ± SD				
Absenteeism	23.5 ± 28.8	18.1 ± 27.8	24.0 ± 33.4	24.6 ± 34.2
Presenteeism	50.0 ± 24.1	44.6 ± 24.4	43.7 ± 24.0	44.5 ± 22.7
Work productivity loss	59.5 ± 27.5	51.5 ± 29.4	56.0 ± 29.7	54.4 ± 30.0
Activity impairment	52.1 ± 23.2	50.4 ± 23.4	54.1 ± 25.8	56.8 ± 24.2
IBDQ score, mean ± SD				
Total score	114 ± 32.4	119 ± 30.5	118 ± 33.1	112 ± 32.1
Bowel symptoms	3.59 ± 1.01	3.67 ± 0.917	3.61 ± 0.974	3.45 ± 0.947
Systemic symptoms	3.39 ± 1.16	3.49 ± 1.09	3.47 ± 1.22	3.25 ± 1.10
Emotional function	3.66 ± 1.10	3.84 ± 1.08	3.89 ± 1.20	3.69 ± 1.16
Social function	3.52 ± 1.37	3.74 ± 1.40	3.64 ± 1.51	3.48 ± 1.41

^a^Other not shown.

^b^Use of corticosteroids or immunomodulators, but not both.

EQ-5D, EuroQol 5-dimension; EQ-5D-5L, EuroQol 5-dimension 5-level; hsCRP, high-sensitivity C-reactive protein; IBDQ, Inflammatory Bowel Disease Questionnaire; MCS, mental component summary; PCS, physical component summary; SD, standard deviation; SF-36, 36-Item Short-Form Survey; UC, ulcerative colitis; VAS, visual analogue scale; WPAI, Work Productivity and Activity Impairment questionnaire.

### 3.2. Primary HRQoL analyses

#### 3.2.1. SF-36

**Figure 1.**
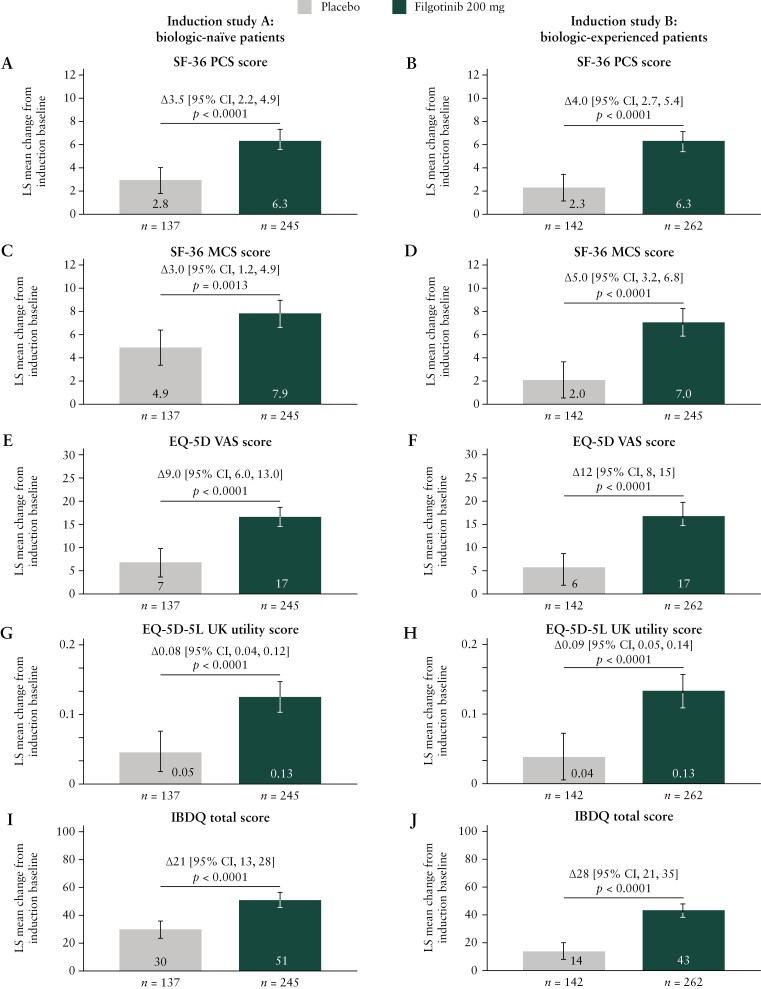
Change in SF-36 PCS [A, B], SF-36 MCS [C, D], EQ-5D VAS [E, F], EQ-5D-5L UK utility [G, H] and IBDQ total [I, J] scores in Induction Studies A and B at week 10. Error bars indicate 95% CIs. CI, confidence interval; EQ-5D, EuroQol 5-dimension; EQ-5D-5L, EuroQol 5-dimension 5-level; IBDQ, Inflammatory Bowel Disease Questionnaire; LS, least-squares; MCS, mental component summary; PCS, physical component.

At week 10, patients who had received filgotinib 200 mg had greater LS mean increases from induction baseline in SF-36 PCS and MCS scores than those who had received placebo [*p* < 0.01] [Figure 1A–D]. Treatment differences were numerically larger among biologic-experienced than biologic-naïve patients. Similar results were observed for all SF-36 subscales [Supplementary Figure 1A and B]. A treatment effect of filgotinib 200 mg compared with placebo was observed on the proportion of patients achieving the MCID in SF-36 PCS score among both biologic-naïve [*p* = 0.0006] and biologic-experienced [*p* < 0.0001] patients, and on the proportion of patients achieving the MCID in SF-36 MCS score among biologic-experienced patients [*p* < 0.0001] [Table 2].

**Table 2.** Proportions of patients achieving the MCID in SF-36, EQ-5D, WPAI and IBDQ total scores in Induction Studies A and B at week 10 and in the Maintenance Study at week 58

	Induction Study A: biologic-naïve patients			Induction Study B: biologic-experienced patients			Maintenance Study		
	Placebo [*n* = 137]	Filgotinib 200 mg [*n* = 245]	Treatment difference, % [95% CI] *p* value	Placebo [*n* = 142]	Filgotinib 200 mg [*n* = 262]	Treatment difference, % [95% CI] *p* value	Placebo [*n* = 98]	Filgotinib 200 mg [*n* = 199]	Treatment difference, % [95% CI] *p* value
**Generic HRQoL**									
SF-36 PCS score, *n* [%]	58 [42.3]	150 [61.2]	18.9 [8.0, 29.7] *p* = 0.0006	45 [31.7]	146 [55.7]	24.0 [13.8, 34.3] *p* < 0.0001	32 [32.7]	122 [61.3]	28.7 [16.4, 40.9] *p* < 0.0001
SF-36 MCS score, *n* [%]	61 [44.5]	133 [54.3]	9.8 [−1.2, 20.7] *p* = 0.0848	41 [28.9]	136 [51.9]	23.0 [12.9, 33.2] *p* < 0.0001	23 [23.5]	103 [51.8]	28.3 [16.6, 39.9] *p* < 0.0001
EQ-5D VAS score, *n* [%]	49 [35.8]	135 [55.1]	19.3 [8.6, 30.1] *p* = 0.0004	41 [28.9]	136 [51.9]	23.0 [12.9, 33.2] *p* < 0.0001	27 [27.6]	107 [53.8]	26.2 [14.2, 38.2] *p* < 0.0001
EQ-5D-5L UK utility score, *n* [%]	56 [40.9]	127 [51.8]	11.0 [0.1, 21.9] *p* = 0.0512	41 [28.9]	137 [52.3]	23.4 [13.3, 33.6] *p* < 0.0001	24 [24.5]	102 [51.3]	26.8. [15.0, 38.5] *p* < 0.0001
**Work productivity**									
WPAI activity impairment score, *n* [%]	78 [56.9]	172 [70.2]	13.3 [2.6, 23.9] *p* = 0.0123	64 [45.1]	166 [63.4]	18.3 [7.7, 28.9] *p* = 0.0006	29 [29.6]	123 [61.8]	32.2 [20.2, 43.3] *p* < 0.0001
WPAI absenteeism score, *n*/*N*^a^ [%]	29/83 [34.9]	49/138 [35.5]	0.6 [−13.0, 14.1] *p* = 0.9999	21/81 [25.9]	46/155 [29.7]	3.8 [−9.1, 16.6] *p* = 0.6492	9/60 [15.0]	28/110 [25.5]	10.5 [−3.0, 23.9] *p* = 0.1663
WPAI presenteeism score, *n*/*N*^a^ [%]	39/83 [47.0]	81/138 [58.7]	11.7 [−2.8, 26.2] *p* = 0.1205	26/81 [32.1]	69/155 [44.5]	12.4 [−1.4, 26.2] *p* = 0.0878	12/60 [20.0]	59/110 [53.6]	33.6 [18.6, 48.7] *p* < 0.0001
WPAI work productivity loss score, *n*/*N*^a^ [%]	46/83 [55.4]	85/138 [61.6]	6.2 [−8.2, 20.6] *p* = 0.4454	36/81 [44.4]	85/155 [54.8]	10.4 [−3.9, 24.7] *p* = 0.1677	15/60 [25.0]	62/110 [56.4]	31.4 [15.7, 47.0] *p* = 0.0002
**Disease-specific HRQoL**									
IBDQ total score, *n* [%]	83 [60.6]	193 [78.8]	18.2 [8.0, 28.4] *p* = 0.0002	52 [36.6]	180 [68.7]	32.1 [21.8, 42.3] *p* < 0.0001	32 [32.7]	136 [68.3]	35.7 [23.6, 47.8] *p* < 0.0001
**Combined HRQoL outcomes**									
Combined IBDQ, and SF-36 PCS and MCS scores, *n* [%]	36 [26.3]	98 [40.0]	13.7 [3.6, 23.9] *p* = 0.0098	19 [13.4]	95 [36.3]	22.9 [14.3, 31.5] *p* < 0.0001	19 [19.4]	93 [46.7]	27.3 [16.1, 38.6] *p* < 0.0001

^a^ *N* indicates the number of patients in employment at induction baseline.

CI, confidence interval; EQ-5D, EuroQol 5-dimension; EQ-5D-5L, EuroQol 5-dimension 5-level; HRQoL, health-related quality of life; IBDQ, Inflammatory Bowel Disease Questionnaire; MCID, minimal clinically important difference; SF-36, 36-Item Short-Form Survey; VAS, visual analogue scale; WPAI, Work Productivity and Activity Impairment questionnaire.

**Figure 2.**
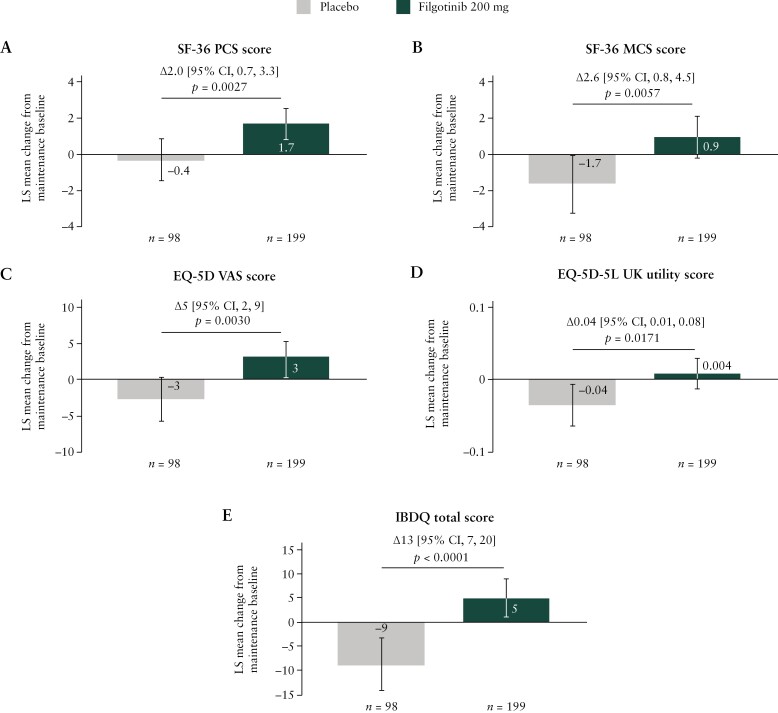
Change in SF-36 PCS [A], SF-36 MCS [B], EQ-5D VAS [C], EQ-5D-5L UK utility [D] and IBDQ total [E] scores in the Maintenance Study at week 58. Error bars indicate 95% CIs. CI, confidence interval; EQ-5D, EuroQol 5-dimension; EQ-5D-5L, EuroQol 5-dimension 5-level; IBDQ, Inflammatory Bowel Disease Questionnaire; LS, least-squares; MCS, mental component summary; PCS, physical component summary; SF-36, 36-Item Short-Form Survey; VAS, visual analogue scale.

At week 58, patients in the filgotinib 200 mg group had LS mean increases from maintenance baseline in SF-36 PCS and MCS scores, whereas patients in the respective placebo group had LS mean decreases in the same measures [*p* < 0.01] [Figure 2A and B]. A treatment difference was reported across all SF-36 subscales except for physical functioning [all *p* < 0.05] [Supplementary Figure 1C]. Greater proportions of filgotinib 200 mg- than placebo-treated patients achieved the MCIDs in SF-36 PCS and MCS scores [both *p* < 0.0001] [Table 2].

In a further analysis, we assessed restoration of HRQoL as measured using SF-36 scores. Among patients with an SF-36 PCS or MCS score of <40 at induction baseline, a greater proportion of those treated with filgotinib 200 mg than those treated with placebo achieved restoration of SF-36-defined HRQoL [SF-36 PCS or MCS score ≥40] by week 10 in the biologic-naïve and biologic-experienced populations [all *p* < 0.05] [Supplementary Table 2]. At week 58, normal HRQoL was maintained in numerically higher proportions of patients treated with filgotinib 200 mg compared with placebo, though the differences in proportions were not nominally significant.

#### 3.2.2. EQ-5D

At week 10, filgotinib 200 mg-treated patients had a greater LS mean increase from induction baseline in EQ-5D VAS and EQ-5D-5L UK utility scores than placebo-treated patients, in both the biologic-naïve and biologic-experienced populations [all *p* < 0.0001] [Figure 1E–H]. A treatment effect of filgotinib 200 mg compared with placebo on the proportion of patients who achieved the MCID in EQ-5D VAS was observed in both patient populations [biologic-naïve, *p* = 0.0004; biologic-experienced, *p* < 0.0001], and on the proportion of patients who achieved the MCID in EQ-5D-5L UK utility score in the biologic-experienced population [*p* < 0.0001] [Table 2].

At week 58, patients in the filgotinib 200 mg group experienced LS mean increases from maintenance baseline in EQ-5D VAS and EQ-5D-5L UK utility scores, whereas patients in the respective placebo group experienced LS mean decreases [EQ-5D VAS, *p* = 0.0030; EQ-5D-5L UK utility, *p* = 0.0171] [Figure 2C and D]. In addition, greater proportions of filgotinib 200 mg-treated than placebo-treated patients achieved the MCID in both EQ-5D measures [both *p* < 0.0001] [Table 2].

#### 3.2.3. WPAI

Greater proportions of patients who received filgotinib 200 mg than those who received placebo achieved the MCID in WPAI activity impairment score at week 10 [biologic-naïve, *p* = 0.0123; biologic-experienced, *p* = 0.0006] and at week 58 [*p* < 0.0001] [Table 2]. Among patients who were employed at induction baseline, greater proportions of those treated with filgotinib 200 mg than those treated with placebo achieved the MCID for both presenteeism [*p* < 0.0001] and work productivity loss [*p* = 0.0002] scores at week 58.

#### 3.2.4. IBDQ

At week 10, filgotinib 200 mg-treated patients had a greater LS mean increase from induction baseline in IBDQ total score than placebo-treated patients (biologic-naïve: 51 vs 30 points, Δ 21 [95% CI, 13, 28], *p* < 0.0001; biologic-experienced: 43 vs 14 points, Δ 28 [95% CI, 21, 35], *p* < 0.0001) [Figure 1I and J]. Similar results were observed for each of the four IBDQ subscales [Supplementary Figure 2]. In addition, greater proportions of filgotinib 200 mg-treated than placebo-treated patients achieved the MCID in IBDQ total score at week 10 [biologic-naïve, *p* = 0.0002; biologic-experienced, *p* < 0.0001] [Table 2].

At week 58, patients in the filgotinib 200 mg group experienced an LS mean increase from maintenance baseline in IBDQ total score of 5 points, compared with a 9-point decrease in patients in the respective placebo group [*p* < 0.0001] [Figure 2E]. Similar results were observed for each of the four IBDQ subscales [Supplementary Figure 3]. In addition, greater proportions of patients in the maintenance filgotinib 200 mg group than the respective ­placebo group achieved the MCID in IBDQ total score [*p* < 0.0001] [Table 2].

IBDQ remission was achieved by greater proportions of filgotinib 200 mg-treated than placebo-treated patients at week 10 [biologic-naïve, 55.9% vs 35.0%; biologic-experienced, 43.1% vs 17.6%; both *p* < 0.0001] and week 58 [57.8% vs 25.5%, *p* < 0.0001] [Supplementary Figure 4].

### 3.3. Secondary HRQoL analyses

#### 3.3.1. Proportions of patients achieving CDC

To assess the treatment benefits of filgotinib 200 mg in individuals, we evaluated the proportions of patients who achieved the exploratory composite endpoint, CDC [comprising pMCS remission, endoscopic improvement, inflammatory biomarker remission and IBDQ remission] at weeks 10 and 58. In the overall induction population, CDC was achieved by greater proportions of filgotinib 200 mg-treated than placebo-treated patients [10.9% vs 2.9%, *p* < 0.001]. A treatment difference was observed among biologic-naïve patients [*p* < 0.001], but not among biologic-experienced patients [Table 3]. Treatment differences for filgotinib 200 mg compared with placebo at week 10 were also observed for each of the individual CDC components [pMCS remission, endoscopic improvement, inflammatory biomarker remission and IBDQ remission] among biologic-naïve and biologic-experienced patients [all *p* < 0.05]. At week 58, greater proportions of filgotinib 200 mg-treated than placebo-treated patients achieved CDC [22.1% vs 7.1%, *p* = 0.002] and each CDC component [all *p* < 0.05] except for inflammatory biomarker remission [Table 3].

**Table 3.** Proportions of patients achieving CDC and its component outcomes in Induction Studies A and B at week 10 and in the Maintenance Study at week 58

	Placebo	Filgotinib 200 mg	Treatment difference, % [95% CI] *p* value
**Overall induction, *n***	**277** ^a^	**505** ^a^	
CDC,^b^*n* [%]	8 [2.9]	55 [10.9]	8.0 [4.4, 11.6] *p* < 0.001
pMCS remission,^c^*n* [%]	55 [19.9]	218 [43.2]	23.3 [16.7, 30] *p* < 0.001
Endoscopic improvement,^d^*n* [%]	42 [15.2]	128 [25.3]	10.1 [4.2, 16.1] *p* = 0.001
Inflammatory biomarker remission,^e^*n* [%]	37 [13.4]	152 [30.2]	16.8 [10.8, 22.7] *p* < 0.001
IBDQ remission,^f^*n* [%]	75 [27.1]	253 [50.1]	23.0 [15.9, 30.1] *p* < 0.001
**Induction Study A: biologic-naïve patients, *n***	**136** ^a^	**245**	
CDC,^b^*n* [%]	6 [4.4]	43 [17.6]	13.2 [6.7, 19.6] *p* < 0.001
pMCS remission,^c^*n* [%]	43 [31.6]	132 [53.9]	22.3 [11.7, 32.8] *p* < 0.001
Endoscopic improvement,^d^*n* [%]	30 [22.1]	83 [33.9]	11.8 [2.1, 21.5] *p* = 0.021
Inflammatory biomarker remission,^e^*n* [%]	28 [20.6]	102 [41.6]	21.0 [11.3, 30.8] *p* < 0.001
IBDQ remission,^f^*n* [%]	49 [36.0]	137 [55.9]	19.9 [9.1, 30.6] *p* < 0.001
**Induction Study B: biologic-experienced patients, *n***	**141** ^a^	**260** ^a^	
CDC,^b^*n* [%]	2 [1.4]	12 [4.6]	3.2 [−0.6, 7.0] *p* = 0.167
pMCS remission,^c^*n* [%]	12 [8.5]	86 [33.1]	24.6 [16.7, 32.5] *p* < 0.001
Endoscopic improvement,^d^*n* [%]	12 [8.5]	45 [17.3]	8.8 [1.7, 15.9] *p* = 0.024
Inflammatory biomarker remission,^e^*n* [%]	9 [6.4]	50 [19.2]	12.8 [6.0, 19.7] *p* = 0.001
IBDQ remission,^f^*n* [%]	26 [18.4]	116 [44.6]	26.2 [16.8, 35.5] *p* < 0.001
**Maintenance Study, *n***	**98**	**199**	
CDC,^b^*n* [%]	7 [7.1]	44 [22.1]	15.0 [6.5, 23.4] *p* = 0.002
pMCS remission,^c^*n* [%]	26 [26.5]	123 [61.8]	35.3 [23.5, 47.1] *p* < 0.001
Endoscopic improvement,^d^*n* [%]	15 [15.3]	81 [40.7]	25.4 [14.8, 36] *p* < 0.001
Inflammatory biomarker remission,^e^*n* [%]	44 [44.9]	88 [44.2]	−0.7 [−13.4, 12] *p* = 0.990
IBDQ remission,^f^*n* [%]	55 [56.1]	143 [71.9]	15.8 [3.3, 28.1] *p* = 0.010

^a^Excluding four patients from the full analysis set without HRQoL data.

^b^CDC was defined as achievement of pMCS remission, endoscopic improvement, inflammatory biomarker remission and IBDQ remission.

^c^pMCS remission was defined as a pMCS ≤2 and no individual rectal bleeding, stool frequency or physician’s global assessment subscore >1.

^d^Endoscopic improvement was defined as a Mayo endoscopic subscore of 0 or 1.

^e^Inflammatory biomarker remission was defined as faecal calprotectin <150 µg/g.^19^.

^f^IBDQ remission was defined as an IBDQ total score ≥170.

CDC, Comprehensive Disease Control; CI, confidence interval; HRQoL, health-related quality of life; IBDQ, Inflammatory Bowel Disease Questionnaire; pMCS, partial Mayo Clinic Score.

#### 3.3.2. Proportions of patients experiencing minimal clinically important improvements and declines in HRQoL and work productivity measures by CDC achievement

To evaluate the association between CDC achievement and HRQoL, we assessed clinically important improvements from induction baseline to week 10, and clinically important declines from maintenance baseline to week 58, in generic HRQoL and work productivity outcomes. Greater proportions of patients who achieved CDC at week 10 than those who did not achieve CDC experienced minimal clinically important improvements from induction baseline in the following measures: SF-36 PCS, SF-36 MCS and all SF-36 subscale scores, EQ-5D VAS and EQ-5D-5L UK utility scores, and WPAI activity impairment score [all *p* < 0.05] [Table 4 and Supplementary Table 3]. Differences were observed among biologic-naïve patients in each of these measures, and among biologic-experienced patients in SF-36 PCS score; in SF-36 physical role limitations, bodily pain, general health perceptions and mental health subscale scores; and in EQ-5D VAS score [all *p* < 0.05]. Among patients who were employed at induction baseline, greater proportions of CDC achievers than non-achievers at week 10 also achieved clinically important improvements from induction baseline in WPAI absenteeism, presenteeism and work productivity loss scores [all *p* < 0.05], with differences observed among biologic-naïve patients [all *p* < 0.05]. At week 58, a lower proportion of CDC achievers than non-achievers experienced clinically important declines from maintenance baseline in SF-36 bodily pain and social functioning subscale scores, and in EQ-5D VAS and WPAI activity impairment scores [all *p* < 0.05] [Table 4 and Supplementary Table 3]. In addition, among patients who were employed at induction baseline, a lower proportion of CDC achievers than non-achievers had a clinically important decline in WPAI presenteeism score [*p* < 0.05].

**Table 4.** Proportions of patients who experienced minimal clinically important improvements from induction baseline to week 10 and minimal clinically important declines from maintenance baseline to week 58 in SF-36, EQ-5D and WPAI scores, among CDC achievers and non-achievers

	MCID threshold	Overall induction			Induction Study A: biologic-naïve patients			Induction Study B: biologic-experienced patients			Maintenance Study		
		CDC achievers [*n* = 63]	CDC non-achievers [*n* = 699]	Treatment difference, % [95% CI] *p* value	CDC achievers [*n* = 49]	CDC non-achievers[*n* = 325]	Treatment difference, % [95% CI] *p* value	CDC achievers [*n* = 14]	CDC non-achievers [*n* = 374]	Treatment difference, % [95% CI] *p* value	CDC achievers [*n* = 51]	CDC non-achievers [*n* = 246]	Treatment difference, % [95% CI] *p* value
**Generic HRQoL**													
SF-36 PCS score, *n* [%]	3.8	51 [81.0]	354 [50.6]	30.3 [19.1, 41.6] *p* < 0.001	38 [77.6]	173 [53.2]	24.3 [10.3, 38.4] *p* = 0.001	13 [92.9]	181 [48.4]	44.5 [26.3, 62.6] *p* = 0.002	2 [3.9]	33 [13.4]	−9.5 [−17.5, −1.5] *p* = 0.058
SF-36 MCS score, *n* [%]	4.6	47 [74.6]	330 [47.2]	27.4 [15.2, 39.6] *p* < 0.001	38 [77.6]	158 [48.6]	28.9 [14.9, 43.0] *p* < 0.001	9 [64.3]	172 [46.0]	18.3 [−11.0, 47.6] *p* = 0.275	8 [15.7]	53 [21.5]	−9.5 [−17.5, −1.5] *p* = 0.346
EQ-5D VAS score, *n* [%]	Improvement: 10.9; Decline: −14.4	48 [76.2]	318 [45.5]	30.7 [18.7, 42.7] *p* < 0.001	37 [75.5]	150 [46.2]	29.4 [15.0, 43.7] *p* < 0.001	11 [78.6]	168 [44.9]	33.7 [7.9, 59.4] *p* = 0.015	0 [0.0]	38 [15.5]	−15.4 [−21.1, −9.7] *p* = 0.001
EQ-5D-5L UK utility score, *n* [%]	Improvement: 0.076; Decline: −0.109	48 [76.2]	317 [45.4]	30.8 [18.8, 42.9] *p* < 0.001	38 [77.6]	147 [45.2]	32.3 [18.3, 46.4] *p* < 0.001	10 [71.4]	170 [45.5]	26.0 [−1.9, 53.9] *p* = 0.098	6 [11.8]	58 [23.6]	−11.8 [−23.3, −0.3] *p* = 0.062
**Work productivity**													
WPAI activity impairment score, *n* [%]	7%	56 [88.9]	424 [60.7]	28.2 [18.8, 37.7] *p* < 0.001	45 [91.8]	205 [63.1]	28.8 [18.3, 39.2] *p* < 0.001	11 [78.6]	219 [58.6]	20.0 [−5.8, 45.8] *p* = 0.171	7 [13.7]	70 [28.5]	−14.7 [−26.9, −2.5] *p* = 0.029
WPAI absenteeism score, *n*/*N*^a^ [%]	7%	19/39 [48.7]	126/418 [30.1]	18.6 [0.9, 36.3] *p* = 0.017	16/31 [51.6]	62/190 [32.6]	19.0 [−1.7, 39.7] *p* = 0.040	3/8 [37.5]	64/228 [28.1]	9.4 [−31.1, 49.9] *p* = 0.691	2/39 [5.1]	15/189 [7.9]	−2.8 [−12.3, 6.7] *p* = 0.744
WPAI presenteeism score, *n*/*N*^a^ [%]	7%	26/39 [66.7]	189/418 [45.2]	21.4 [4.5, 38.4] *p* = 0.010	22/31 [71.0]	98/190 [51.6]	19.4 [0.0, 38.8] *p* = 0.045	4/8 [50.0]	91/228 [39.9]	10.1 [−31.6, 51.8] *p* = 0.717	1/39 [2.6]	28/189 [14.8]	−12.2 [−20.9, −3.6] *p* = 0.035
WPAI work productivity loss score, *n*/*N*^a^ [%]	7%	36/39 [92.3]	216/418 [51.7]	40.6 [29.6, 51.7] *p* < 0.001	29/31 [93.6]	102/190 [53.7]	39.9 [26.8, 52.9] *p* < 0.001	7/8 [87.5]	114/228 [50.0]	37.5 [7.2, 67.8] *p* = 0.066	2/39 [5.1]	32/189 [16.9]	−11.8 [−22.1, −1.5] *p* = 0.081

^a^ *N* indicates the number of patients employed at induction baseline.

CDC was defined as achievement of pMCS remission, endoscopic improvement, inflammatory biomarker remission and IBDQ remission.

CDC, Comprehensive Disease Control; EQ-5D, EuroQol 5-dimension; EQ-5D-5L, EuroQol 5-dimension 5-level; HRQoL, health-related quality of life; IBDQ, Inflammatory Bowel Disease Questionnaire; MCID, minimal clinically important difference; MCS, mental component summary; PCS, physical component summary; pMCS, partial Mayo Clinic Score; SF-36, 36-Item Short-Form Survey; VAS, visual analogue scale, WPAI, Work Productivity and Activity Impairment questionnaire.

#### 3.3.3. Proportions of patients in histological remission among CDC achievers vs non-achievers

**Figure 3.**
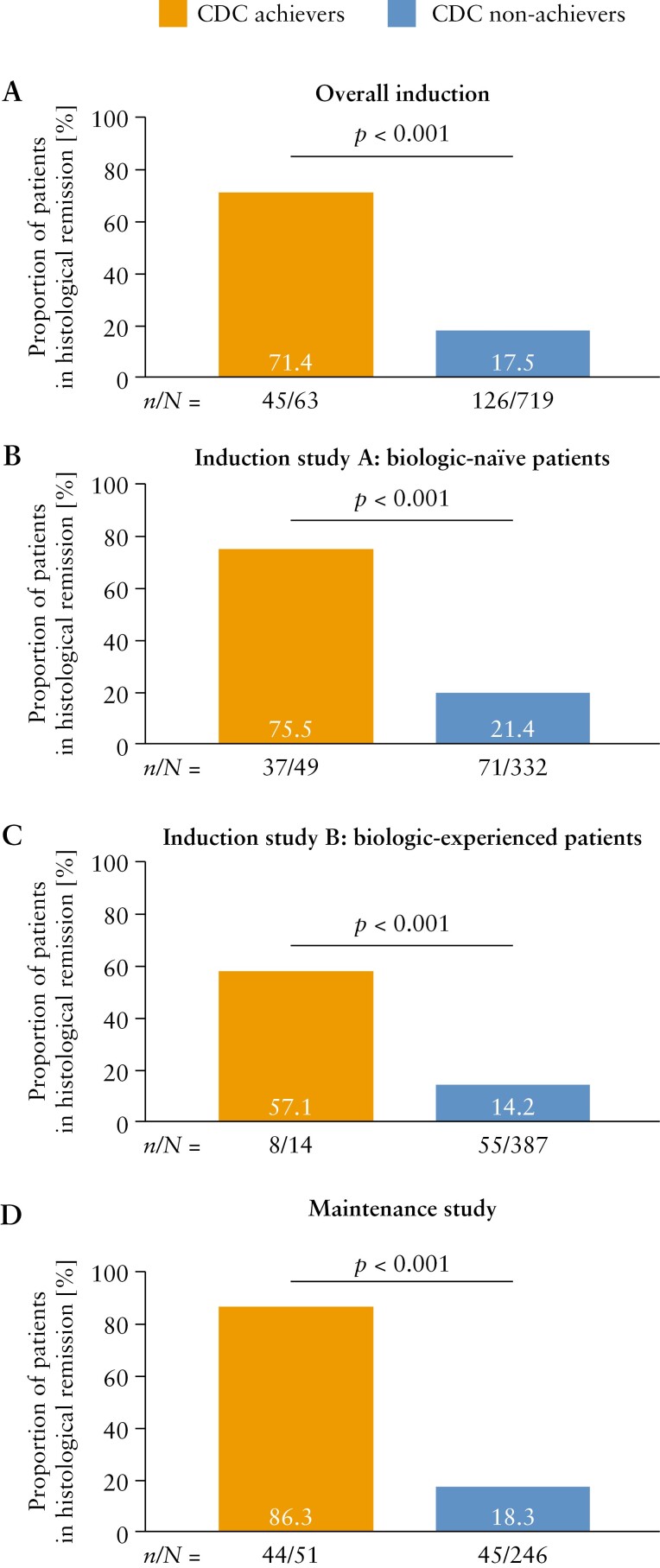
Proportions of CDC achievers and non-achievers in histological remission in Induction Studies A and B [A–C] at week 10, and in the Maintenance Study at week 58 [D]. CDC was defined as achievement of pMCS remission, endoscopic improvement, inflammatory biomarker remission and IBDQ remission. Histological remission was defined as a grade 0 Geboes score of ≤0.3, grade 1 score of ≤1.1, grade 2A score of ≤2A.3, grade 2B score of 2B.0, grade 3 score of 3.0, grade 4 score of 4.0 and grade 5 score of 5.0. CDC, Comprehensive Disease Control; IBDQ, Inflammatory Bowel Disease Questionnaire; pMCS, partial Mayo Clinic Score.

To assess the relationship between achievement of CDC and histomorphology [as an objective marker of disease activity], the proportions of patients who did and did not achieve CDC who were in histological remission were investigated. At week 10, greater proportions of CDC achievers than non-achievers were in histological remission [biologic-naïve: 75.5% vs 21.4%; biologic-experienced: 57.1% vs 14.2%; both *p* < 0.001] [Figure 3]. In addition, at week 58, greater proportions of patients who achieved CDC were in histological remission, compared with those who did not achieve CDC [86.3% vs 18.3%, *p* < 0.001].

## 4. Discussion

This study assessed the effects of treatment with filgotinib on generic and disease-specific HRQoL, and work productivity in patients with UC in the SELECTION trial. Moreover, to examine the effect of filgotinib at the patient level, we developed and evaluated a four-component composite endpoint, CDC. Treatment with filgotinib 200 mg resulted in improvements in generic and disease-specific HRQoL in the short term [10 weeks] among both biologic-naïve and biologic-experienced patients, with improvements maintained during long-term treatment [58 weeks]. In addition, filgotinib 200 mg treatment was effective in restoring patients’ HRQoL as measured by SF-36 scores to that of the general population after 10 weeks of treatment.

Patients consider the ability to improve HRQoL to be among the most important attributes of a UC treatment.^10^ This is in part due to the positive association between improved HRQoL and symptomatic remission. Previous work by our group has demonstrated a moderate correlation between IBDQ remission and various metrics of gut disease activity, including pMCS remission (74.6% [induction phase] and 83.7% [maintenance phase] concordance with IBDQ remission), a rectal bleeding subscore of 0 (71.9% [induction] and 83.7% [maintenance] concordance) and stool frequency subscore of ≤1 (72.8% [induction] and 83.9% [maintenance] concordance).^20^ Whilst this finding does underscore the relationship between the different outcomes, it also suggests that there are additional dimensions to the effect of disease on HRQoL outside of those relating to gut dysfunction.

Restoration of HRQoL is now among the long-term treatment targets recommended for UC.^11^ Notably, patients treated with filgotinib 200 mg in SELECTION experienced considerable improvements across the broad range of HRQoL measures included in these analyses [SF-36, EQ-5D and IBDQ] during both induction and maintenance, vs placebo. When considering the proportion of patients demonstrating restoration of overall HRQoL (determined as SF-36 PCS or MCS score ≥40) this generally improved following treatment with filgotinib. This reached nominal significance relative to placebo in the induction phase (*p* < 0.05), whereas in the maintenance phase it did not. A possible explanation for this could be that participants who were randomized to receive placebo in the maintenance phase had already received filgotinib in the induction phase and were in response and/or remission at the beginning of the maintenance phase. Indeed, analysis of disease worsening revealed that of patients who received filgotinib 200 mg in the induction phase before being withdrawn to placebo in the maintenance phase, only 50% demonstrated significant disease worsening during the maintenance phase, suggesting that filgotinib still exerted some therapeutic effects 58 weeks later (data unpublished at present).

Treatment differences in HRQoL improvements at week 10 were numerically larger in biologic-experienced than biologic-naïve patients, despite the former group having a lower response rate [53.1% and 66.5% of biologic-experienced and biologic-naïve filgotinib 200 mg-treated patients achieved an MCS response at week 10, respectively^15]^. This may reflect the finding that biologic-experienced patients had more severe disease and thus a greater unmet treatment need than biologic-naïve patients [77.8% and 55.8% had an induction baseline Mayo endoscopic subscore of 3, respectively], and therefore perceived any treatment benefits to be greater. In addition, the benefit of filgotinib 200 mg on WPAI outcomes provides evidence that approximately a quarter of patients had improvements in health status that allowed them to return to their normal working lives. Induction treatment with filgotinib 200 mg was also shown to restore HRQoL after 10 weeks compared with placebo in a proportion of patients with low induction baseline SF-36 scores.

To assess the benefits of filgotinib 200 mg at the patient level, we combined endpoints that are treatment targets specified by the STRIDE-II guidelines,^11^ and that are typically reported as part of independent cross-sectional analyses. A greater proportion of patients treated with filgotinib 200 mg than those treated with placebo simultaneously achieved each of the four CDC components [pMCS remission, endoscopic improvement, inflammatory biomarker remission and IBDQ remission], at both week 10 and week 58. The cut-off of faecal calprotectin 150 µg/g was chosen because any value above this level is considered to be indicative of active disease, and associated with an increased risk of disease flares.^19^ The proportion of patients who achieved CDC was lower than the proportion of those who achieved each of the individual components. This indicates that the combination of the four outcomes [clinical, endoscopic, biomarker and patient-reported] was difficult to attain. Importantly, CDC was achieved in parallel with short- and long-term improvements in HRQoL, as shown by clinically important improvements in SF-36 and EQ-5D scores at week 10 that were sustained in high proportions of CDC achievers by week 58. This result suggests that achievement of CDC may be associated with restoration of HRQoL. In addition, achievement of CDC was associated with histological remission, a gold standard morphological measure of disease activity,^21^ indicating that patients who achieved the endpoint may have had controlled disease at the cellular level. Indeed, previous analyses by our group have demonstrated a significant association between histological remission and endoscopic improvement, a well-established clinical endpoint (data not shown). Further work, including prospective trials and assessment of risk–benefit profiles in patient subpopulations, is required to validate our exploratory composite endpoint. Nevertheless, these initial observations point to the potential of assessing multiple established outcomes within individuals as an approach for measuring multidimensional disease control that relates to the patient’s overall health status.

Limitations of these analyses include that HRQoL measures were assessed at only two time points. Future studies could assess individual patient trajectories using multiple endpoint data at various time points [from the beginning of treatment through to CDC], in order to evaluate disease burden and fluctuations over time, and to determine early indicators of a positive/negative disease course. A further limitation was that only responders entered the Maintenance Study, owing to the re-randomization trial design. This introduces bias due to the exclusion of non-responders. Nevertheless, this was not considered a major issue, as the purpose of the maintenance phase was to evaluate the perpetuation of previously identified therapeutic benefit arising from the induction phase. It is also worth noting that for the group who received induction filgotinib and maintenance placebo, as noted above, it cannot be ruled out that priming with filgotinib impacted the disease trajectory during maintenance when filgotinib treatment ceased. However, if anything this is likely to favour an underestimation of the benefits of filgotinib. In addition, this is a study design that is frequently employed in UC trials as it allows for evaluation of different treatment durations whilst minimizing the time that any individual patients spend on the placebo. This is important as withholding of favourable care would present ethical issues.

Analyses evaluating the composite endpoint were exploratory and require formal external validation, for example reliability testing. This is particularly true considering the response to filgotinib of inflammatory biomarkers. These are well-established indicators of active disease, as well as being the least invasive of all the objective endpoints, and the lack of concordance of their profiles with other disease markers warrants further investigation. Alternatively, our data could suggest the threshold for inflammatory biomarker remission could be made less stringent, given that clinically significant improvements to HRQoL and endoscopic improvement were achieved in this study without the criterion for inflammatory biomarker remission being met. Nevertheless, it could be speculated that assessment of multiple established UC outcomes within a composite endpoint may be valuable for clinicians managing patients with UC, as treatment shifts towards a more personalized and patient-centred approach.^22,23^ Such composite efficacy measures assessing disease control within individuals could fuel a new generation of algorithm trials that help guide decisions on the sequential use of targeted therapies, and could aid in cost-effectiveness evaluations.

### 4.1. Conclusions

Based upon data from the SELECTION trial, filgotinib 200 mg resulted in short- and long-term improvements in generic and disease-specific HRQoL. Filgotinib 200 mg led to some patients achieving a stringent exploratory composite endpoint that may be associated with both improved HRQoL and histological remission. Further work is required to validate this exploratory endpoint.

## Supplementary Material

jjad018_suppl_Supplementary_Materials

## Data Availability

Anonymized individual patient data will be shared upon request for research purposes dependent upon the nature of the request, the merit of the proposed research, the availability of the data and its intended use. The full data sharing policy for Gilead Sciences, Inc., can be found at https://www.gileadclinicaltrials.com/transparency-policy/.

## Notes

Part of this work was presented at the European Crohn’s and Colitis Organisation (ECCO) 2022 congress (virtual congress).
