# Supplementary material for: Filgotinib Improved Health-Related Quality of Life and Led to Comprehensive Disease Control in Individuals with Ulcerative Colitis: Data from the SELECTION Trial
Source: J Crohns Colitis. 2023 Jun 16;17(6):863–75. doi: 10.1093/ecco-jcc/jjad018 (PMC10274306; doi:10.1093/ecco-jcc/jjad018)
Supplement: jjad018_suppl_Supplementary_Materials [file jjad018_suppl_supplementary_materials.docx]

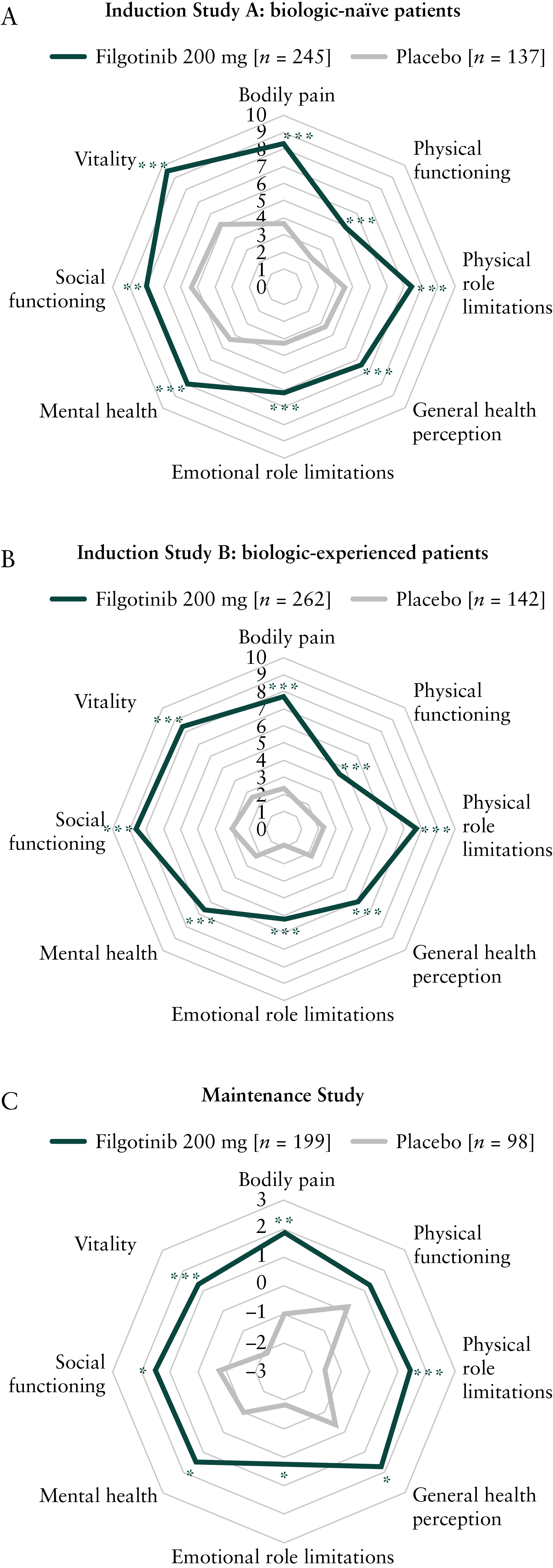
**Supplementary figures and tables**


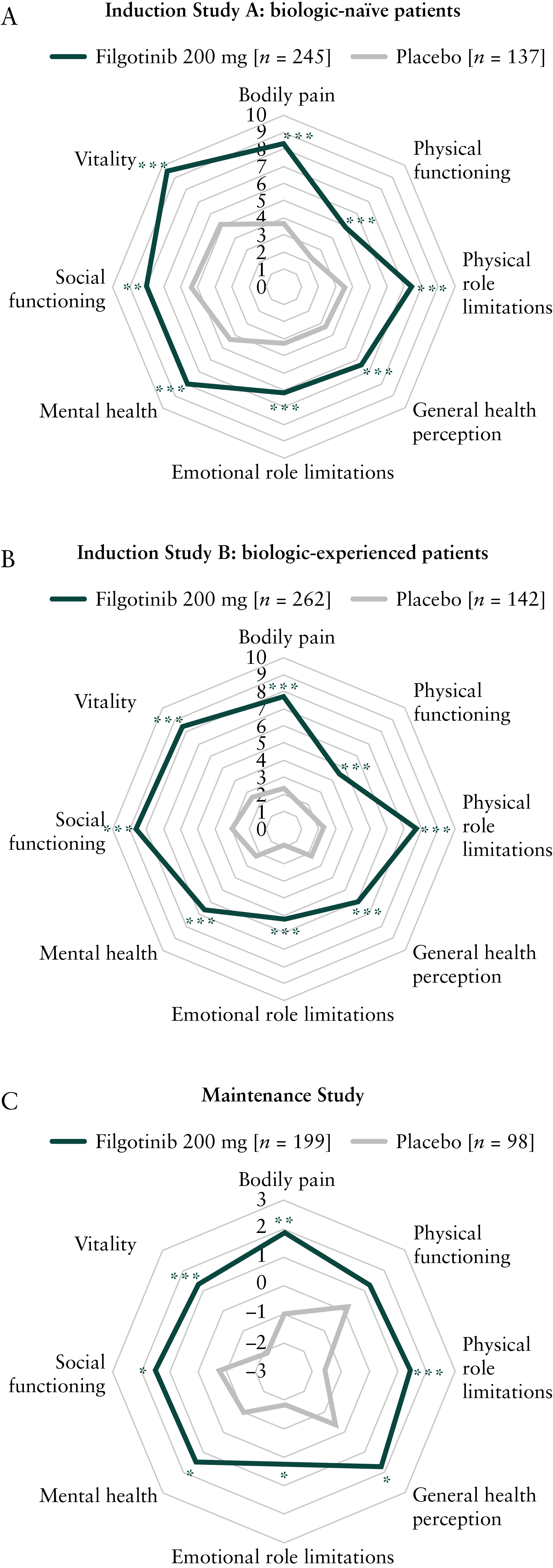


**
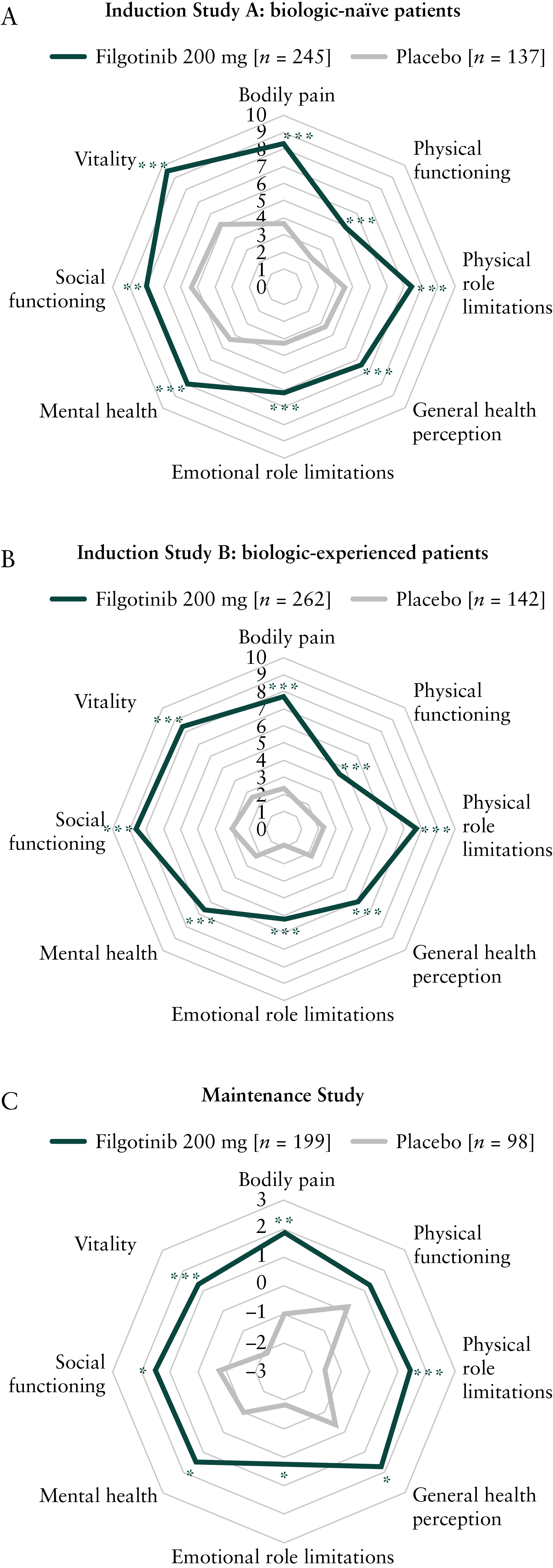
**

**Supplementary Figure 1.** Change in SF-36 subscale scores in Induction Studies A and B [A, B] at week 10, and in the Maintenance Study at week 58 [C]. **p* < 0.05 filgotinib 200 mg vs placebo. ***p* < 0.01 filgotinib 200 mg vs placebo. ****p* < 0.001 filgotinib 200 mg vs placebo. Concentric octagons represent the LS mean change from induction baseline [A, B] or maintenance baseline [C] in the SF-36 subscale score. LS, least-squares; SF-36, 36-Item Short-Form Survey.


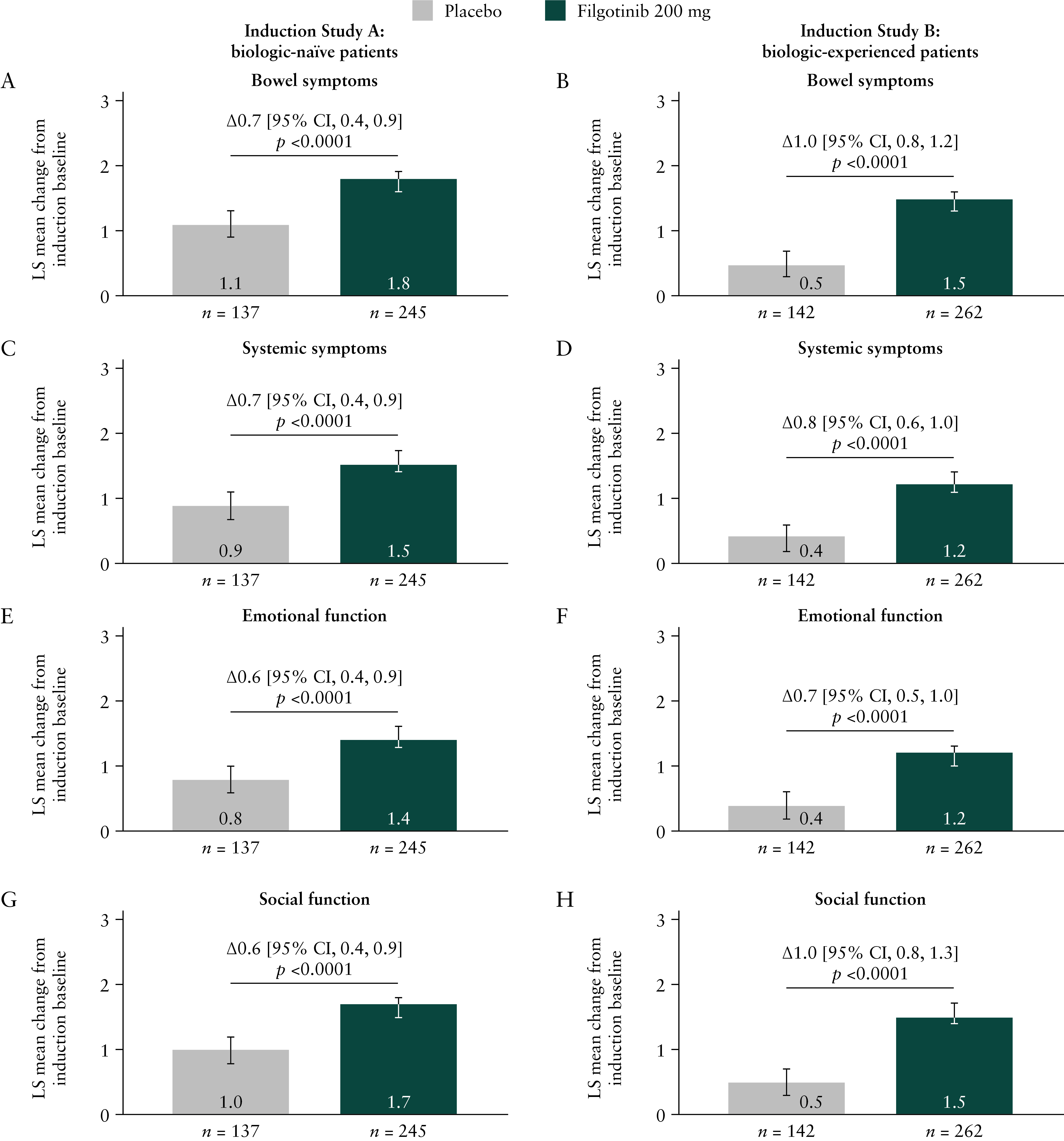


**Supplementary Figure 2.** Change in IBDQ bowel symptoms [A, B], systemic symptoms [C, D], emotional function [E, F] and social function [G, H] subscale scores in Induction Studies A and B at week 10. Error bars indicate 95% CIs. CI, confidence interval; IBDQ, Inflammatory Bowel Disease Questionnaire; LS, least-squares.


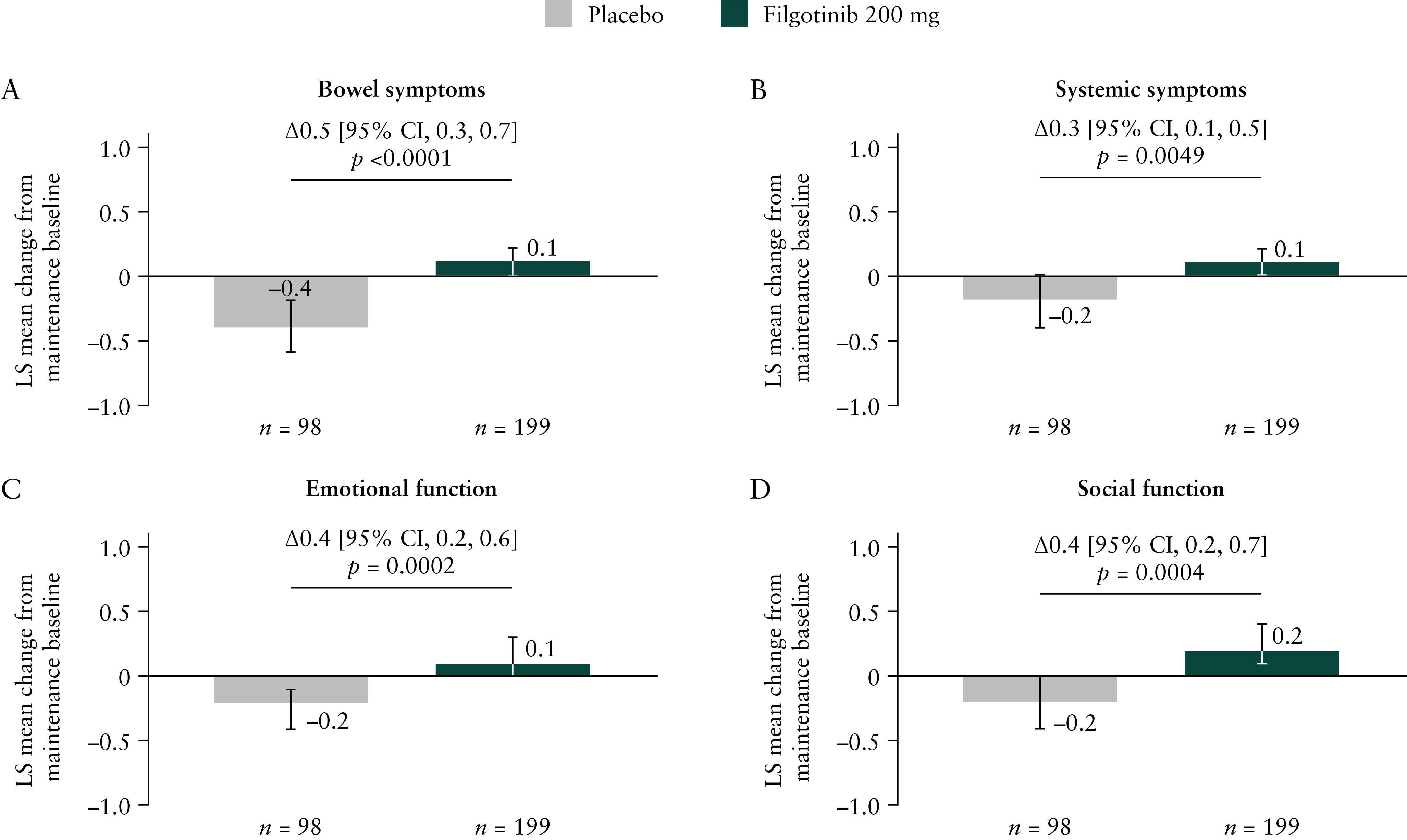


**Supplementary Figure 3.** Change in IBDQ bowel symptoms [A], systemic symptoms [B], emotional function [C] and social function [D] subscale scores in the Maintenance Study at week 58. Error bars indicate 95% CIs. CI, confidence interval; IBDQ, Inflammatory Bowel Disease Questionnaire; LS, least-squares.


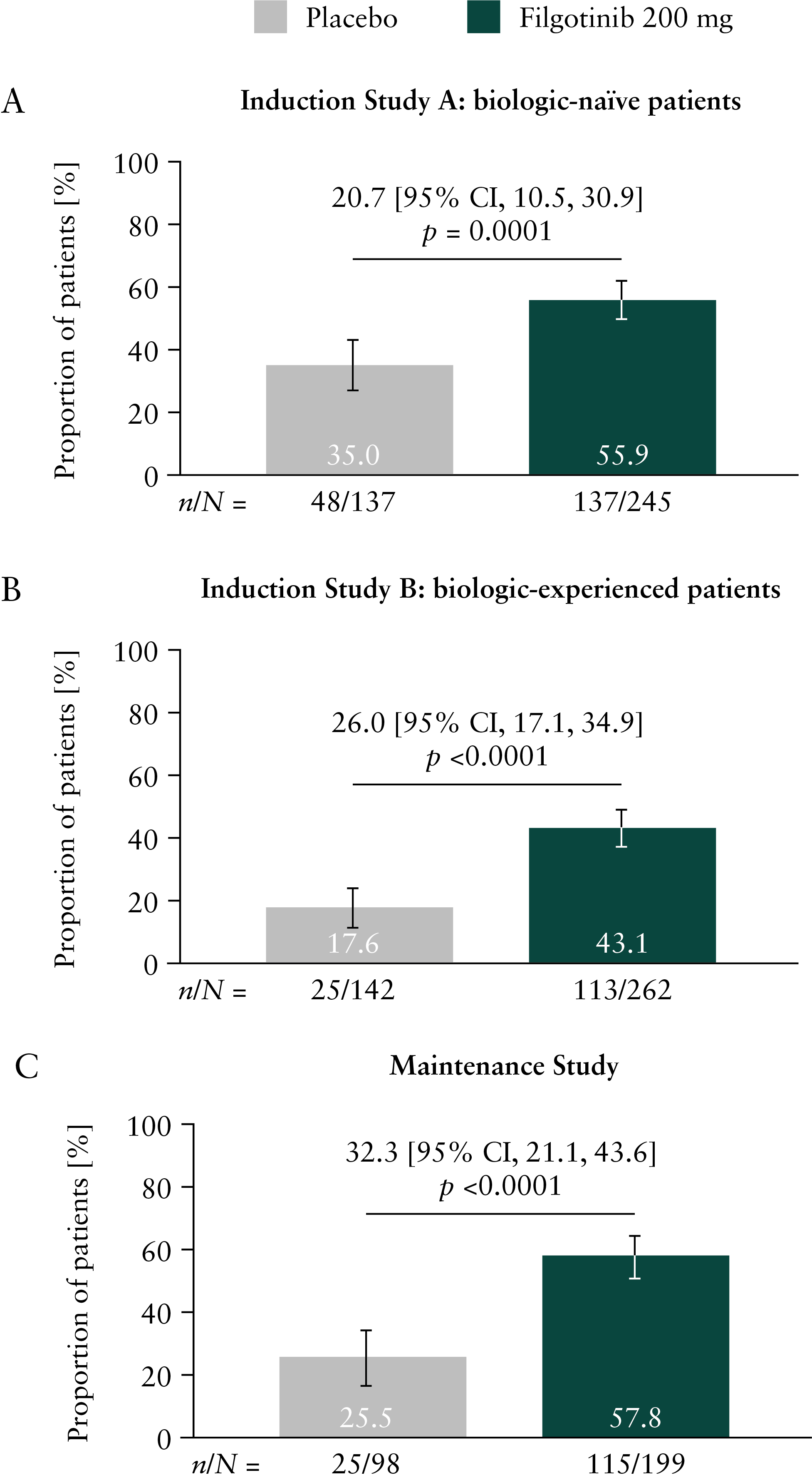


**Supplementary Figure 4.** Proportions of patients who achieved IBDQ remission in Induction Studies A and B [A, B] at week 10, and in the Maintenance Study at week 58 [C]. IBDQ remission was defined as an IBDQ total score ≥170. Error bars indicate 95% CIs. CI, confidence interval; IBDQ, Inflammatory Bowel Disease Questionnaire.

**Supplementary Table 1.** Description of HRQoL instruments

| **Instrument** | **Scores, scales and domains** | **Score ranges** | **MCID thresholds** |
| --- | --- | --- | --- |
| **SF-36 v2**^18^ | **PCS score** | Normative based scores adjusted for age and sex: mean average of 50 and standard deviation of 10  Higher scores demonstrate better HRQoL | **PCS score: 3.8** |
|  | ·Physical functioning |  | Physical functioning: 4.3 |
|  | ·Physical role limitations |  | Physical role limitations: 4.0 |
|  | ·Bodily pain |  | Bodily pain: 5.5 |
|  | ·General health perceptions |  | General health perceptions: 7.0 |
|  | **MCS score** |  | **MCS score: 4.6** |
|  | ·Energy/vitality |  | Energy/vitality: 6.7 |
|  | ·Social functioning |  | Social functioning: 6.2 |
|  | ·Emotional role limitations |  | Emotional role limitations: 4.6 |
|  | ·Mental health |  | Mental health: 6.7 |
| **EQ-5D**^16,17,23^ | **VAS score** | VAS: 0–100 | **Improvement:** |
|  | **5L UK utility score** | 5L UK utility: 0–1^a^  Higher scores demonstrate better health utility | VAS: 10.9 |
|  | ·Mobility |  | 5L UK utility: 0.076 |
|  | ·Self-care |  | **Decline:** |
|  | ·Usual activities |  | VAS: −14.4 |
|  | ·Pain/discomfort |  | 5L UK utility: −0.109 |
|  | ·Anxiety/depression |  |  |
| **WPAI**^24^ | ·Presenteeism | Six-item survey  Percentage  Lower scores indicate less impairment | **7-point change** |
|  | ·Absenteeism |  |  |
|  | ·Work impairment |  |  |
|  | ·Activity impairment |  |  |
| **IBDQ v5**^25–27^ | **Total score** | Total score: 32–224  Subscales: 1–7  Higher total score demonstrates better HRQoL, and higher subscale scores demonstrate better function | **16-point change in IBDQ total score**  MCID not available for IBDQ subscales  IBDQ total score ≥170 indicates IBDQ remission |
|  | ·Bowel systems [10 items] |  |  |
|  | ·Emotional health [12 items] |  |  |
|  | ·Systemic systems [5 items] |  |  |
|  | ·Social function [5 items] |  |  |
|  |  |  |  |
|  |  |  |  |
|  |  |  |  |
|  |  |  |  |

^a^Theoretical range, actual utilities may be >1 or <0.

EQ-5D, EuroQol 5-dimension; EQ-5D-5L, EuroQol 5-dimension 5-level; HRQoL, health-related quality of life; IBDQ, Inflammatory Bowel Disease Questionnaire; MCID, minimal clinically important difference; MCS, mental component summary; PCS, physical component summary; VAS, visual analogue scale; WPAI, Work Productivity and Activity Impairment questionnaire.

**Supplementary Table 2.** Restoration of SF-36-defined HRQoL at weeks 10 and 58

|  | **ITT,  *n*** | **Excluded^a^** | ***n*/*N*^b^ [%]** | **Odds ratio  [95% CI]** | ***p* value** |  |
| --- | --- | --- | --- | --- | --- | --- |
| **SF-36 PCS score ≥40** | | | | | | |
| **Overall induction** | |  | | | |  |
| Filgotinib 200 mg | 507 | 306 | 126/201 [62.7] | 2.71 [1.69, 4.35] | < 0.001 | |
| Placebo | 279 | 164 | 44/115 [38.3] | Reference |  |  |
| **Induction Study A: biologic-naïve patients** | |  | | | |  |
| Filgotinib 200 mg | 245 | 162 | 59/83 [71.1] | 2.25 [1.07, 4.76] | 0.033 | |
| Placebo | 137 | 91 | 24/46 [52.2] | Reference |  |  |
| **Induction Study B: biologic-experienced patients** | |  | | | |  |
| Filgotinib 200 mg | 262 | 144 | 67/118 [56.8] | 3.22 [1.71, 6.07] | < 0.001 | |
| Placebo | 142 | 73 | 20/69 [29] | Reference |  |  |
| **Maintenance Study** | |  | | | |  |
| Filgotinib 200 mg | 199 | 19 | 174/180 [96.7] | 2.05 [0.64, 6.54] | 0.226 | |
| Placebo | 98 | 7 | 85/91 [93.4] | Reference |  |  |
| **SF-36 MCS score ≥40** | | | | | | |
| **Overall induction** | |  | | | |  |
| Filgotinib 200 mg | 507 | 238 | 157/269 [58.4] | 2.15 [1.43, 3.22] | < 0.001 | |
| Placebo | 279 | 127 | 60/152 [39.5] | Reference |  |  |
| **Induction Study A: biologic-naïve patients** | |  | | | |  |
| Filgotinib 200 mg | 245 | 120 | 82/125 [65.6] | 2.54 [1.44, 4.49] | 0.001 | |
| Placebo | 137 | 53 | 36/84 [42.9] | Reference |  |  |
| **Induction Study B: biologic-experienced patients** | |  | | | |  |
| Filgotinib 200 mg | 262 | 118 | 75/144 [52.1] | 1.99 [1.10, 3.61] | 0.023 | |
| Placebo | 142 | 74 | 24/68 [35.3] | Reference |  |  |
| **Maintenance Study** | |  | | | |  |
| Filgotinib 200 mg | 199 | 36 | 148/163 [90.8] | 1.81 [0.82, 4.00] | 0.145 | |
| Placebo | 98 | 14 | 71/84 [84.5] | Reference |  |  |

^a^Patients with an SF-36 PCS or MCS score >40 at induction baseline for induction, or an SF-36 PCS or MCS score <40 at maintenance baseline for maintenance.

^b^*n* is the number of patients who achieved SF-36 PCS or MCS ≥40, and *N* is the number of the patients in the ITT population minus those who were excluded.

CI, confidence interval; HRQoL, health-related quality of life; ITT, intention-to-treat; MCS, mental component summary; PCS, physical component summary; SF-36, 36-Item Short-Form Survey.

**Supplementary Table 3.** Proportions of patients who experienced minimal clinically important improvements from induction baseline to week 10 and minimal clinically important declines from maintenance baseline to week 58 in SF-36 subscale scores, among CDC achievers and non-achievers

|  |  | **Overall induction** | | | **Induction Study A:**  **biologic-naïve patients** | | | **Induction Study A:**  **biologic-naïve patients** | | | **Maintenance Study** | | |
| --- | --- | --- | --- | --- | --- | --- | --- | --- | --- | --- | --- | --- | --- |
| **SF-36 subscale score** | **MCID  threshold** | **CDC achievers [*n =* 63]** | **CDC non- achievers [*n =* 699]** | **Treatment difference, % [95% CI]**  ***p* value** | **CDC achievers [*n =* 49]** | **CDC non- achievers [*n =* 325]** | **Treatment difference, % [95% CI]**  ***p* value** | **CDC achievers [*n =* 14]** | **CDC non- achievers [*n =* 374]** | **Treatment difference, % [95% CI]**  ***p* value** | **CDC achievers [*n =* 51]** | **CDC non- achievers [*n =* 246]** | **Treatment difference, % [95% CI]**  ***p* value** |
| Physical functioning, *n* [%] | 4.3 | 36  [57.1] | 251 [35.9] | 21.2  [7.6, 34.8] *p* = 0.001 | 32 [65.3] | 121 [37.2] | 28.1  [12.6, 43.6] *p* < 0.001 | 4 [28.6] | 130 [34.8] | −6.2  [−34, 21.7] *p* = 0.779 | 2 [3.9] | 22 [8.9] | −5  [−12.6, 2.6] *p* = 0.394 |
| Physical role limitations, *n* [%] | 4.0 | 52  [82.5] | 377 [53.9] | 28.6  [17.7, 39.5] *p* < 0.001 | 40 [81.6] | 183 [56.3] | 25.3  [12, 38.6] *p* = 0.001 | 12 [85.7] | 194 [51.9] | 33.8  [11.1, 56.6] *p* = 0.014 | 7 [13.7] | 49 [19.9] | −6.2  [−18.1, 5.7] *p* = 0.303 |
| Bodily pain, *n* [%] | 5.5 | 50  [79.4] | 321 [45.9] | 33.4  [21.9, 45] *p* < 0.001 | 39 [79.6] | 154 [47.4] | 32.2  [18.5, 45.9] *p* < 0.001 | 11 [78.6] | 167 [44.7] | 33.9  [8.1, 59.7] *p* = 0.014 | 3 [5.9] | 46 [18.7] | −12.8  [−22.1, −3.5] *p* = 0.023 |
| General health perceptions, *n* [%] | 7.0 | 38  [60.3] | 248 [35.5] | 24.8  [11.4, 38.3] *p* < 0.001 | 28 [57.1] | 121 [37.2] | 19.9  [3.9, 35.9] *p* = 0.008 | 10 [71.4] | 127 [34.0] | 37.5  [9.6, 65.3] *p* = 0.008 | 5 [9.8] | 34 [13.8] | −4  [−14.4, 6.4] *p* = 0.648 |
| Energy/vitality,  *n* [%] | 6.7 | 44  [69.8] | 288 [41.2] | 28.6  [15.9, 41.4] *p* < 0.001 | 35 [71.4] | 140 [43.1] | 28.3  [13.4, 43.3] *p* < 0.001 | 9 [64.3] | 148 [39.6] | 24.7  [−4.6, 54] *p* = 0.094 | 4 [7.8] | 43 [17.5] | −9.6  [−19.6, 0.3] *p* = 0.095 |
| Social functioning, *n* [%] | 6.2 | 40  [63.5] | 295 [42.2] | 21.3  [8, 34.6]  *p* < 0.001 | 30 [61.2] | 134 [41.2] | 20  [4.2, 35.8]  *p* = 0.009 | 10 [71.4] | 161 [43.1] | 28.4  [0.5, 56.3] *p* = 0.052 | 1 [2.0] | 29 [11.8] | −9.8  [−16.6, −3.1] *p* = 0.038 |
| Emotional role limitations, *n* [%] | 4.6 | 35  [55.6] | 281 [40.2] | 15.4  [1.7, 29]  *p* = 0.018 | 29 [59.2] | 138 [42.5] | 16.7  [0.8, 32.7] *p* = 0.028 | 6 [42.9] | 143 [38.2] | 4.6  [−25.5, 34.7] *p* = 0.727 | 5 [9.8] | 44 [17.9] | −8.1  [−18.7, 2.6] *p* = 0.213 |
| Mental health,  *n* [%] | 6.7 | 45  [71.4] | 275 [39.3] | 32.1  [19.5, 44.7] *p* < 0.001 | 36 [73.5] | 139 [42.8] | 30.7  [16, 45.4] *p* < 0.001 | 9 [64.3] | 136 [36.4] | 27.9  [−1.4, 57.2] *p* = 0.047 | 5 [9.8] | 35 [14.2] | −4.4  [−14.9, 6] *p* = 0.503 |

CDC was defined as achievement of pMCS remission, endoscopic improvement, inflammatory biomarker remission and IBDQ remission.

CDC, Comprehensive Disease Control; IBDQ, Inflammatory Bowel Disease Questionnaire; MCID, minimal clinically important difference; pMCS, partial Mayo Clinic Score; SF-36, 36-Item Short-Form Survey.
